# Secondary mutant ALK-I1171s in pituitary metastases from a patient with ALK fusion-positive advanced lung adenocarcinoma: A case report and literature review

**DOI:** 10.3389/fonc.2022.1016320

**Published:** 2022-10-17

**Authors:** Dan Han, Kewei Zhao, Qin Yang, Liling Zhang, Shihong Fei

**Affiliations:** Cancer Center, Union Hospital, Tongji Medical College, Huazhong University of Science and Technology, Wuhan, China

**Keywords:** pituitary metastasis, crizotinib, drug resistance, ALK, lung adenocarcinoma

## Abstract

**Background:**

Pituitary metastasis accounts for a very low percentage of cases of brain metastasis from lung cancer, and there are uncertainties and challenges in diagnosis and treatment. We hope to shed some light on the diagnosis and treatment by reporting a case of ALK fusion mutation-positive lung cancer pituitary metastasis.

**Case presentation:**

We report a 48-year-old female patient with an initial diagnosis of stage IVB lung adenocarcinoma with ALK fusion. The patient developed headache, dizziness, hypopituitarism and hyperprolactinemia one year after treatment with crizotinib. Later, the patient underwent neurosurgical resection of the pituitary tumor and then symptomatic relief. Postoperative pathology suggested pituitary metastasis, and the next-generation gene sequencing conducted on the pituitary metastasis indicated that secondary drug resistance mutation ALK-I1171s occurred after the ALK fusion gene.

**Conclusion:**

In this article, we present a patient with suspected pituitary metastases with lung cancer. The progression to pituitary mass resection and next-generation gene sequencing of the pituitary metastasis are suggestive for further diagnosis and treatment.

## Introduction

According to available reports, pituitary metastases from lung cancer are very rare, especially when compared with metastases to other endocrine glands, such as the adrenal gland and thyroid gland (1). Lung cancer (24.2%) is the second most common malignant tumor for pituitary metastases after breast cancer (37.2%), followed by prostate cancer (5%), kidney cancer (5%) and lymphoma (2). Some studies have found that the average overall survival of patients with pituitary metastases is 10-13 months (3, 4). Patients with pituitary metastases from lung cancer are usually asymptomatic at first. However, as the tumor grows to a certain size, the mass will cause central nervous system and endocrine system symptoms. The most common symptoms are visual impairment, followed by hypopituitarism, hyperprolactinemia, and diabetes insipidus (5). Clinical symptoms and imaging examination cannot effectively differentiate pituitary metastases from benign pituitary tumors. Chemotherapy, radiation therapy, hormonal therapy and surgery are the available treatment options, but there is no conclusive evidence that these treatments can prolong the survival of patients (6). This paper reports an ALK fusion mutation-positive lung adenocarcinoma patient with pituitary metastasis. After surgical treatment, the symptoms of dizziness, headache, hypopituitarism and hyperprolactinemia were significantly relieved. Meanwhile, our team also conducted next-generation gene sequencing of the pituitary metastasis, and the results indicated that secondary drug resistance mutation ALK-I1171s occurred in the ALK fusion gene.

## Case presentation

**Figure 1:**
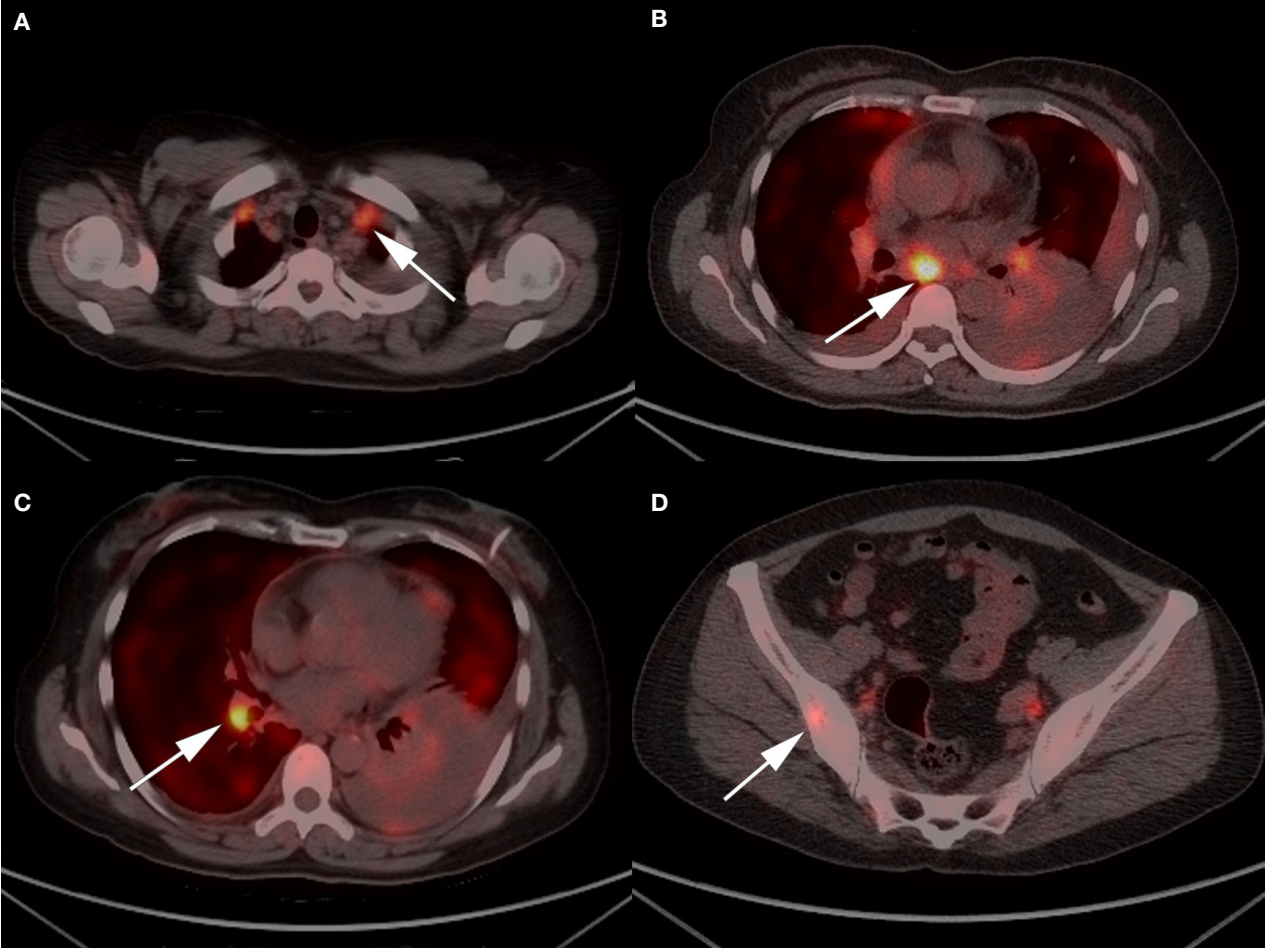
Representative ^18^F/FDG PET-CT images of the patient: **(A)** enlarged bilateral supraclavicular and **(B)** mediastinal lymph nodes; **(C)** a 3.4*2.4 cm soft tissue density mass in the right lower lobe; and **(D)** increased metabolism in right ilium.

**Figure 2:**
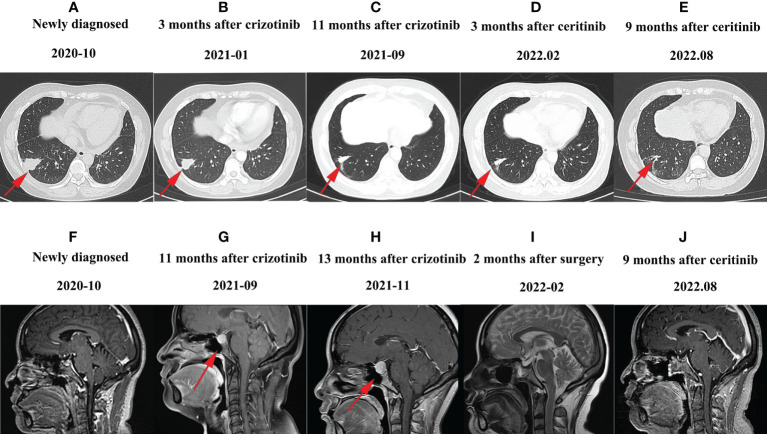
Representative CT or MRI images of the patient: **(A)** CT image of October 2020 before the treatment. **(B)** CT image of January 2021 after treatment with crizotinib. The therapeutic effect of the patients was evaluated as partial response. **(C)** CT image of September 2021 after pituitary metastases; the lung lesion was stable. **(D)** CT image of February 2022 after treatment with ceritinib; the lung lesions shrank further. **(E)** CT image of August 2022 after treatment with ceritinib; the lung lesion is still shrinking. **(F)** MRI image obtained in October 2020 before treatment; brain MRI showed no abnormalities. **(G)** MRI image of September 2021 after finding pituitary metastases. **(H)** MRI image after 1 month of supplemental hormone therapy. The pituitary gland was enlarged. **(I)** MRI image after sphenoid sinus exploration and nasal endoscopic saddle area tumor excision, and the brain MRI showed no mass. **(J)** Follow-up MRI images 9 months after tumor resection and 9 months after ceritinib administration.

**Figure 3:**
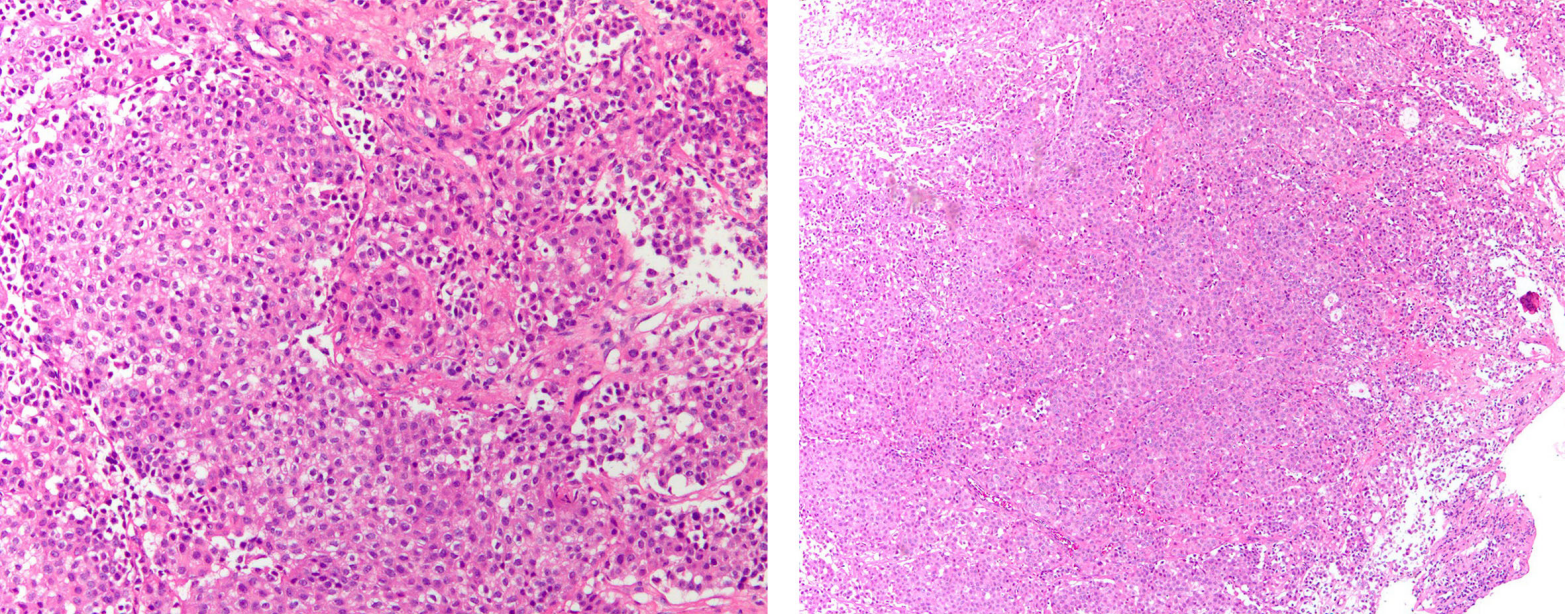
Histopathological findings of pericardial effusion by paraffin embedding of cell sediment. IHC yielded the following: PCK (+), Claudin-4(+), TTF-1(+), NapsinA(+), Calretinin (–), CK5/6 (–), P40 (–), EGFR (–), C-met (–), Ros-1 (–), ALK(+).

**Figure 4:**
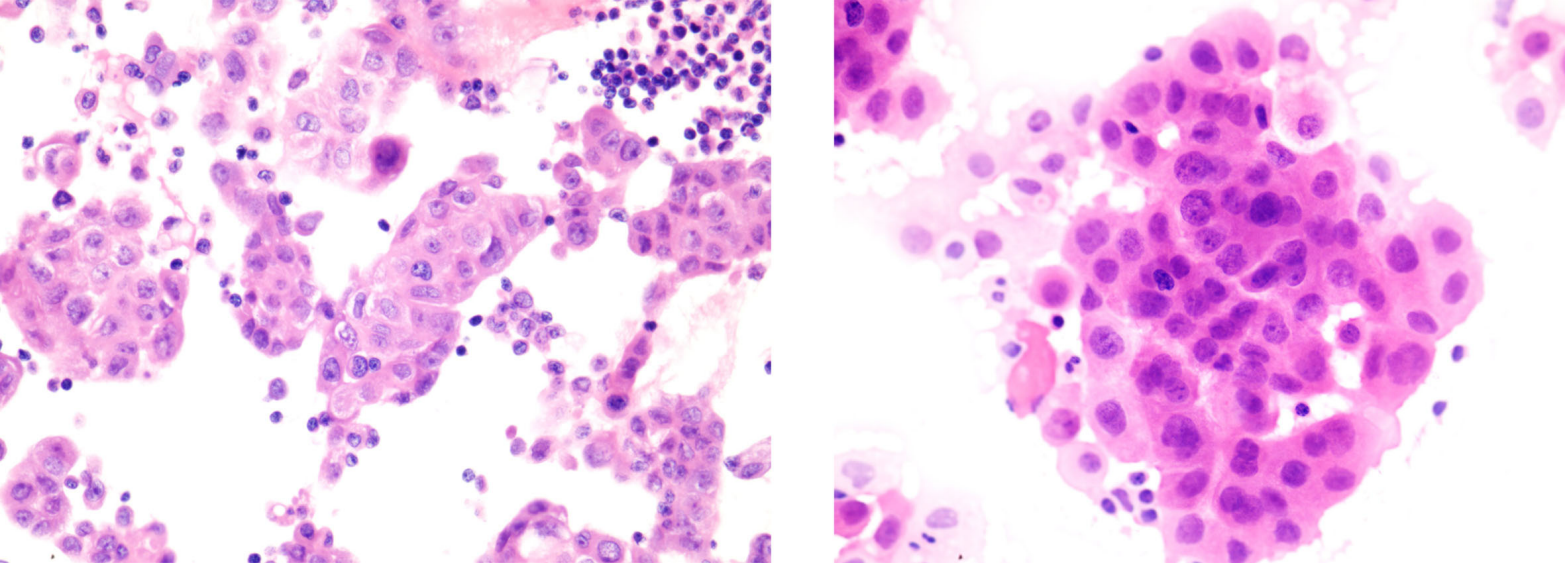
Histopathological findings of pituitary metastases. IHC yielded the following: PCK (+), TTF-1 (+), NapsinA (+), EMA (+), Syn (partial +), CgA (focal +), GFAP (–), S1001 (-), CD68 (-), CD34 (-), ACTH (-), GH (-), PRL (-), FSH (-), LH (-), TSH (-), Ki67 (Li:5%).

A 48-year-old Chinese woman with no smoking history presented to the Union Hospital of Tongji Medical College of Huazhong University of Science and Technology in October 2020 for chest tightness and chest pain. The ^18^F-fluorodeoxyglucose positron emission tomography/computed tomography (^18^F-FDG PET/CT) revealed a soft tissue density shadow in the right lower lobe with a size of 3.4*2.4 cm; enlarged bilateral supraclavicular and mediastinal lymph nodes, thickened bilateral pleura and increased pericardial effusion were metabolism increased; the metabolism in thoracic vertebrae T4, lumbar vertebrae L1, L5, and right ilium were also increased (**Figure 1**). Baseline lung enhanced CT images are shown in **Figure 2A**. Brain MRI showed no abnormalities (**Figure 2F**). In October 2020, the patient underwent ultrasound-guided pericardiocentesis of pericardial effusion. Upon pathological examination of the pericardial effusion, cancer cells were detected, which was consistent with lung adenocarcinoma (**Figure 3**). Immunohistochemistry yielded the following: PCK (+), TTF-1(+), i+), CK5/6 (–), EGFR (–), C-met (–), Ros-1 (–), ALK (+). Next-generation genetic sequencing (whole-exome sequencing by company of gloriousmed) suggested the ALK EML4 (7, 8) - ALK (9, 10) fusion. Variant allele frequency (VAF) was 3.81%. Another driver mutation was CUX1 (mutation region: chr7: 101821924, 11 exon c.1004delA, p. Asn335fsTer20, VAF 1:00%), and others were somatic mutations of no clinical value, such as INPP4A, MED12, CDC73, CIC, SACS, NOTCH3, ARID2, RPS2, IRS2, INHA. Therefore, the patient was initially diagnosed with lung adenocarcinoma stage cT2N3M1c IVB with EML4-ALK gene fusion (AJCC 8th Edition). She started treatment with crizotinib (p.o. 250 mg bid) in October 2020 for financial reasons. In January 2021, the patient’s lung CT showed that the lesion in the lower lobe of the right lung was 2.2*1.7 cm, and the pericardial effusion was significantly reduced (**Figure 2B**. According to the criteria of resist1.1, the therapeutic effect was evaluated as partial response (PR). The patient started to have headache and dizziness in September 2021, and brain MRI showed that the pituitary gland was full in shape on September 7, 2021, approximately 1.4*1.4*1.3 cm in size, and the lung lesion was stable (**Figures 2C, G**). Then, a lumbar puncture was performed, and no significant abnormalities were seen in the cerebrospinal fluid panel, cerebrospinal fluid biochemistry or cerebrospinal fluid cytology. ACTH (0 am): 5.83 pg/ml, ACTH (8 am): 10.20 pg/ml, ACTH (4 pm): 7.03 pg/ml (normal value: 7-64 pg/ml); cortisol (0 am):7.0μg/L, cortisol (8 am):3.0μg/L, cortisol (4 pm): 1.0μg/L (normal values: 37.0-194 μg/L); prolactin: 113.35 ng/ml (normal values: 1.2-29.9 ng/ml). No abnormalities were seen in sex hormones, thyroid hormone, growth hormone, or insulin-like growth factor. Afterward, our team conducted a multidisciplinary consultation with neurology, neurosurgery, and endocrinology and recommended a follow-up MRI after 1 month of supplemental hormone therapy. However, one month later, the patient’s symptoms of headache and dizziness worsened, and the brain MRI re-examination showed that the pituitary gland was enlarged with a size of approximately 1.8*1.4*1.6 cm. On November 15, 2021 (**Figure 2H**), the patient underwent sphenoid sinus exploration and nasal endoscopic saddle area tumor excision in our neurosurgery department. During the operation, the neurosurgery team found an occupying lesion in the sellar area with high dural tension, approximately 2*1.5 cm in size, pink in color, and tightly adherent to the surrounding tissues. The postoperative pathology showed that it was consistent with metastatic adenocarcinoma with neuroendocrine marker expression in some areas of pulmonary origin. Immunohistochemistry yielded the following: PCK (+), TTF-1 (+), Napsin A (+), ACTH (–), GH (–), PRL (–), FSH (–), LH (–), TSH (–), Ki67 (Li: 5%) (**Figure 4**). The next-generation sequencing (whole-exome sequencing by company of gloriousmed) conducted on the pituitary metastasis suggested EML4 (6)-ALK (10) fusion with VAF of 2.63%. Interestingly, the ALK gene then revealed a missense mutation (mutation region: chr2: 29445213, 22 exon p. Ile1171Ser, VAF: 29.2%). Four non-clinically relevant somatic gene variants (missense mutations) were also detected, namely INPP4A, MAGI2, AMER1, MED12.The patient’s postoperative headache and dizziness were significantly relieved compared to before. The patient’s hormone levels were reviewed on November 29, 2021, and most of the abnormal hormone levels returned to normal. On December 01, 2021, the pituitary gland MRI was reviewed and showed that the pituitary tumor was significantly smaller than before. Based on the patient’s gene sequencing results, we recommended that the patient replace crizotinib with ceritinib. On February 7, 2022, the patient’s lung CT showed that the lesion in the lower lobe of the right lung had shrunk further with a size of 1.8*1.4 cm, and the brain MRI re-examination showed no abnormalities (**Figures 2D, I**). The patient was reexamined every two months by brain MRI and lung CT. The latest reexamination was on August 7, 2022, and no progression was observed (**Figures 2E, J**).

## Discussion

The incidence of pituitary metastases accounts for 1% of the total incidence of pituitary lesions (7), making clinical differentiation of pituitary tumors from pituitary metastases very challenging. Patients with pituitary metastases from lung cancer are often asymptomatic at the initial stage, and many patients are found to have pituitary occupancy at the time of MRI examination (8). Several studies have confirmed that visual impairment, hypopituitarism, and high prolactin levels are the most common clinical manifestations of pituitary metastases (5, 11). Some studies have shown no significant difference in the overall survival of patients between surgical and nonsurgical treatment of pituitary metastases from lung cancer (12). Aida Javanbakht et al. surveyed 289 patients with pituitary metastases at Dewart Medical Center from 1984-2018 and found that 178 (61.6%) patients opted for surgery for treatment (4). The main surgical option currently available is transsphenoidal surgery, but surrounding tissue adhesions and abundant blood supply often make surgery difficult, and the performance of pituitary metastasectomy requires multidisciplinary cooperation and an experienced neurosurgical team (13). Patients undergoing surgery can suffer from secondary hypopituitarism, which requires lifelong hormone replacement therapy (14). In this case, the patient had a remarkable clinical presentation and underwent surgery after multidisciplinary collaboration between the medical oncology team and the neurosurgery team.

The ALK gene has been identified in a variety of solid tumors and is a potent oncogenic driver gene (15). ALK protein is located on the cell membrane with an extracellular receptor region and an intracellular kinase region. Under normal conditions, two ALK proteins are coupled by extracellular ligands to activate signaling pathways and promote cell growth (16). There are three types of mutations in the ALK gene: 1. ALK fusion mutations are the most common, especially EML4-ALK fusion mutations, and the protein can be activated without ligand; 2. Point mutations are less common and occur mainly in the intracellular kinase region; and after the mutation, they continue downstream conveyance of cell growth signals; 3. Amplification mutations are even less common (17). ALK fusion gene mutation frequency accounts for 2-5% of all non-small cell lung cancers worldwide (16, 17). Patients with ALK fusion mutation non-small cell lung cancer can benefit from crizotinib with an objective response rate of 60% and progression-free survival of 8-10 months, but drug resistance occurs within 1-2 years, with central nervous system relapse being the most common reversal (18, 19). However, alectinib has been shown to have longer PFS than crizotinib in multiple phase III clinical trials, and alectinib is now widely used as a first-line therapy, especially in cases of brain metastasis (20). Since the patient had stable lesions elsewhere, our team decided to perform pituitary occupancy resection to clarify the nature of the lesions and to perform next-generation sequencing if the pathology suggested metastatic adenocarcinoma. Thirty-seven percent of crizotinib resistance cases occur due to ALK secondary resistance mutations, which can be classified as ALK kinase region mutations (28%) and ALK fusion gene copy number amplification (9%) (9). In addition, mechanisms associated with crizotinib resistance also include driver gene conversion as well as tumor heterogeneity (10). This patient showed ALK missense mutations after the development of pituitary metastases, and pituitary tissue genetic sequencing suggested the presence of ALK-I1171S. I1171S refers to the conversion of the second nucleotide (3512 nucleotides of full-length ALK) encoding isoleucine ATC to AGC (serine [S]). Similarly, I1171 includes I1171N (isoleucine ATC converts to AAC (asparagine [N])) and I1171T (isoleucine ATC converts to ACC threonine [T]), etc. Currently, it is unclear why this mutation lead to resistance to crizotinib. Further basic and clinical analyses are required to investigate the biology and clinical significance of the I1171 mutation. However, the I1171 mutation was more frequently reported in secondary resistance mutations after alectinib failure than with crizotinib (21–23). In 2014, Ou SH et al. reported that two NSCLC patients with the EML4-ALK variant initially responded to crizotinib and then alectinib but developed acquired resistance with the presence of a mutation in amino acid residue 1171 (I1171N and I1171S, respectively) located in the hydrophobic regulatory spine of ALK kinase (24). I1171 mutations are believed to decrease the stability of the inhibitor through autophosphorylation (25). Gainor et al. found that ceritinib was effective when patients developed the secondary ALK-1171S mutation (26). Importantly, many researchers found that ALK-I1171 mutations mediate resistance to alectinib while generating sensitivity to ceritinib. ALK-I1171 mutations were found to be the second-most common resistance mutations in post-alectinib therapy (12%). Based on the above evidence, we recommended that our patient switch to ceritinib instead of alectinib, and to date, progression-free survival has been 8 months (22, 26).

## Conclusion

Pituitary metastasis from lung cancer is very rare, and surgical treatment could be a positive option. Although crizotinib was chosen for financial reasons in this patient, alectinib has been shown to have longer PFS than crizotinib in multiple phase III clinical trials, and alectinib is now widely used as first-line therapy, especially in brain metastasis. This patient developed pituitary metastasis after crizotinib resistance, and next-generation sequencing suggested the presence of the ALK I1171S mutation. The ALK I1171S mutation has been shown to result in secondary resistance to crizotinib, and ceritinib has proved effective when patients develop the secondary ALK 1171S mutation.

## Data availability statement

The original contributions presented in the study are included in the article/supplementary material. Further inquiries can be directed to the corresponding authors.

## Ethics statement

Written informed consent was obtained from the individual(s) for the publication of any potentially identifiable images or data included in this article.

## Author contributions

SF and DH had the original idea for the article and guided the treatment and management of the patient. DH and KZ wrote the article incorporating the comments from LZ. All authors reviewed and approved the final draft of the article. All authors contributed to the article and approved the submitted version.

## Funding

This work was funded by the National Natural Science Foundation of China (82002825 to DH).

## Conflict of interest

The authors declare that the research was conducted in the absence of any commercial or financial relationships that could be construed as a potential conflict of interest.

## Publisher’s note

All claims expressed in this article are solely those of the authors and do not necessarily represent those of their affiliated organizations, or those of the publisher, the editors and the reviewers. Any product that may be evaluated in this article, or claim that may be made by its manufacturer, is not guaranteed or endorsed by the publisher.
